# White Matter Abnormalities in Anorexia Nervosa: Psychoradiologic Evidence From Meta-Analysis of Diffusion Tensor Imaging Studies Using Tract Based Spatial Statistics

**DOI:** 10.3389/fnins.2020.00159

**Published:** 2020-03-03

**Authors:** Simin Zhang, Weina Wang, Xiaorui Su, Lei Li, Xibiao Yang, Jingkai Su, Qiaoyue Tan, Youjin Zhao, Huaiqiang Sun, Graham J. Kemp, Qiyong Gong, Qiang Yue

**Affiliations:** ^1^Department of Radiology, Huaxi MR Research Center (HMRRC), West China Hospital of Sichuan University, Chengdu, China; ^2^Department of Radiology, West China Hospital of Sichuan University, Chengdu, China; ^3^Liverpool Magnetic Resonance Imaging Centre (LiMRIC) and Institute of Ageing and Chronic Disease, University of Liverpool, Liverpool, United Kingdom

**Keywords:** anorexia nervosa, diffusion tensor, tract-based spatial statistics, fractional anisotropy, magnetic resonance imaging, psychoradiology

## Abstract

**Background:** Anorexia nervosa (AN) is a debilitating illness whose neural basis remains unclear. Studies using tract-based spatial statistics (TBSS) with diffusion tensor imaging (DTI) have demonstrated differences in white matter (WM) microarchitecture in AN, but the findings are inconclusive and controversial.

**Objectives:** To identify the most consistent WM abnormalities among previous TBSS studies of differences in WM microarchitecture in AN.

**Methods:** By systematically searching online databases, a total of 11 datasets were identified, including 245 patients with AN and 246 healthy controls (HC). We used Seed-based d Mapping to analyze fractional anisotropy (FA) differences between AN patients and HC, and performed meta-regression analysis to explore the effects of clinical characteristics on WM abnormalities in AN.

**Results:** The pooled results of all AN patients showed robustly lower FA in the corpus callosum (CC) and the cingulum compared to HC. These two regions preserved significance in the sensitivity analysis as well as in all subgroup analyses. Fiber tracking showed that the WM tracts primarily involved were the body of the CC and the cingulum bundle. Meta-regression analysis revealed that the body mass index and mean age were not linearly correlated with the lower FA.

**Conclusions:** The most consistent WM microstructural differences in AN were in the interhemispheric connections and limbic association fibers. These common “targets” advance our understanding of the complex neural mechanisms underlying the puzzling symptoms of AN, and may help in developing early treatment approaches.

## Introduction

Anorexia nervosa (AN) is a serious mental and somatic disorder that typically develops during adolescence and primarily affects females (Zipfel et al., 2015). With a prevalence of about 0.3% it is relatively rare, but has serious medical consequences leading to death in ~10% of cases, and thus poses a major clinical, psychological and societal burden (Nielsen, 2001). AN is characterized by extreme restriction of energy intake, a distorted body image, excessive concerns over weight and shape, and emotional dysfunction (American Psychiatric Association (APA), 2013; Zipfel et al., 2015). There may be severe long-term medical and psychological sequelae besides acute effects of self-starvation (Steinhausen, 2002). The etiology of AN remains unknown, and the interaction of neurobiological, psychological and environmental factors in its onset and outcome is unclear (Kaye et al., 2013; Zipfel et al., 2015). Exploring the neurobiological abnormalities associated with AN will be important for improving the effectiveness of both diagnosis and treatment (Hill et al., 2016).

With the development of noninvasive neuroimaging technology, diffusion tensor imaging (DTI), as an important psychoradiologic technique (Lui et al., 2016; Kressel, 2017; Port, 2018; Sun et al., 2018; https://radiopaedia.org/articles/psychoradiology), has become a powerful tool for detecting white matter (WM) microstructural differences in various psychiatric illnesses, including schizophrenia (Hao et al., 2006), depression (Kieseppa et al., 2010) as well as bipolar disorder (Wessa et al., 2009). Fractional anisotropy (FA) is the most commonly used DTI metric for exploring anisotropy, quantifying the directionality of diffusion. FA is considered as a highly sensitive but fairly non-specific biomarker of brain WM microstructural architecture and neuropathology (Alexander et al., 2007).

To investigate whole brain FA differences, metrics can be extracted globally by either voxel-based analysis (VBA) or tract-based spatial statistics (TBSS). Several such studies have demonstrated FA differences between patients with AN and healthy controls (HC). Unfortunately, their results are not consistent. Most studies report *lower* FA in widespread WM regions, including the corpus callosum (CC) (Frieling et al., 2012; Frank et al., 2013; Shott et al., 2016; Gaudio et al., 2017; Phillipou et al., 2018; von Schwanenflug et al., 2019), fornix fibers (Kazlouski et al., 2011; Frank et al., 2013; Gaudio et al., 2017), thalamus (Frieling et al., 2012; Hu et al., 2017), cingulum (Kazlouski et al., 2011; Frank et al., 2013), posterior thalamic radiation (PTR) (Phillipou et al., 2018), superior longitudinal fasciculus (SLF) (Via et al., 2014), fronto-occipital fasciculus (FOF) (Kazlouski et al., 2011; Via et al., 2014), corona radiation (Shott et al., 2016; Phillipou et al., 2018) and cerebellum (Nagahara et al., 2014; Shott et al., 2016). Five studies, however, observed *no* significant FA differences between AN patients and HC (Yau et al., 2013; Cha et al., 2016; Pfuhl et al., 2016; Bang et al., 2018; Olivo and Swenne, 2019). Two studies also reported *higher* FA in corona radiation, SLF, FOF, PTR, and CC (Frank et al., 2013; Vogel et al., 2016). These inconsistencies might be due to differences in sample size or in the demographic and clinical characteristics of the patients, and heterogeneity in the imaging protocols. In such situations a powerful way to isolate reliable neurobiological markers is meta-analysis.

To our knowledge, only Barona and colleagues have conducted a coordinate-based meta-analysis of whole-brain DTI studies in AN (Barona et al., 2019). However, the study has a major limitation in using two different methods (TBSS and VBA) to undertake whole brain analysis. VBA is relatively direct, involving spatial normalization of high-resolution images from all subjects to the same stereotactic space (Ashburner and Friston, 2000). By contrast TBSS is a statistical approach, particularly developed to analyze DTI data. It restricts analysis to the center of major WM fibers by projecting every subject's FA data onto the mean skeleton, thus alleviating the misalignment problems that can affect regular VBA. Briefly, TBSS is a more accurate method for exploring disorganization of WM architecture (Smith et al., 2006).

Our aims in this paper are: first, to conduct an updated meta-analysis of TBSS studies to define the most prominent and replicable WM microarchitecture abnormalities in patients with AN using Seed-based d Mapping (SDM), a statistical technique for meta-analyzing studies which use neuroimaging techniques such as fMRI, VBM, DTI or PET to investigate the changes of brain activity or structure (https://www.sdmproject.com/). This method is now widely accepted and has been used in studies of major depressive disorder (Jiang et al., 2017), childhood maltreatment (Lim et al., 2014) and bipolar disorder (Wise et al., 2016). Second, to perform subgroup meta-analyses based on the effects of age and stage of the disorder. Third, to use meta-regression to examine the potential effects of age, illness duration and body mass index (BMI) on the reported WM abnormalities. We hypothesized that AN patients would manifest lower FA compared to HC in tracts involved in reward-related processing (*viz*. CC, fornix, thalamic projections, and striatum) and limbic regions. We also speculated that WM microarchitecture abnormalities might be associated with factors related to starvation (*viz*. decreased BMI and illness duration) and AN symptomatology.

## Materials and Methods

### Literature Search Strategy

We searched for publications on the PubMed, Ovid databases, Web of Science, Science Direct and Google Scholar. The last screen was performed in March 2019. The key search terms were: (“anorexia nervosa” or “eating disorder” or “anorexia”) and (“tract-based spatial statistical” or “TBSS” or “diffusion tensor” or “DTI” or “diffusion tensor imaging” or “fractional anisotropy” or “FA”). The reference lists of identified studies and relevant reviews were manually checked for further studies.

### Selection Criteria and Data Extraction

Studies were included according to the following criteria: (a) articles written in the English language and published in peer-reviewed journals; (b) a primary diagnosis of AN according to the international classification of diseases-10 (ICD-10) and/or Diagnostic and Statistical Manual of Mental Disorders (DSM); (c) studies reported a TBSS comparison between patients with AN and HC; (d) studies detected FA differences at the whole-brain level and reported the results in stereotactic 3D coordinates (Talairach or MNI). When details were not reported in the original manuscripts, a request was made to the corresponding author by e-mail.

Studies were excluded according to the following criteria: (a) meta-analysis, case reports or reviews; (b) studies lacking a HC group; (c) if several studies reported overlapping samples, only the paper reporting the largest sample size was selected. We conducted this meta-analysis according to Preferred Reporting Items for Systematic Reviews and Meta-Analysis (PRISMA) guidelines (Liberati et al., 2009).

The quality of each included study was assessed using a 12-point checklist (see Table S1) that focused on both the clinical and demographic aspects of individual studies and on the imaging methodology (Du et al., 2014). From each included study we recorded first author, cohort size, demographics (age and gender), illness variables (stage of AN, subtype of AN, age at onset, illness duration, BMI, symptom severity), imaging parameters, data processing method and statistical threshold; the peak coordinates were extracted using the SDM tool (Radua et al., 2014b). Two authors (SZ and WW) did this independently, any disagreement being resolved by discussion.

### SDM Meta-Analysis

We conducted a voxel-based analysis to identify brain regions showing consistent significant differences in FA between AN patients and HC, according to the standardized process of the SDM software (http://www.sdmproject.com). Briefly, the SDM tool recreates a map of the effect size based on the peak coordinates extracted from each included study.

The robustness of the main findings was checked by three complementary analyses. First, jack-knife sensitivity analysis was performed to assess the replicability of the results by iteratively repeating the same analysis, discarding a data set each time to establish whether the results remained significant (Radua et al., 2014a). Second, a random-effects model with Q statistics was used to detect the statistical (between-studies) heterogeneity of individual clusters. Third, Egger tests using STATA (www.stata.cn) were used to assess publication bias.

Initially, we planned to perform subgroup meta-analysis of adolescent vs. adult subjects, medicated vs. drug-free subjects, as well as acute cases vs. recovered subjects. However, the number of studies in most of these subgroups (adolescent, medicated, drug-free, recovered) was too small to draw reliable conclusions. Finally, meta-analysis of the subgroups was conducted only for the adult subjects and the acute subjects.

All analytical processes were as described in the SDM tutorial (https://www.sdmproject.com/software/tutorial.pdf) and related publications (Radua et al., 2014b). We adopted the default SDM thresholds (anisotropy = 1.0; full-width at half-maximum = 20 mm, voxel *p* = 0.005, peak height threshold *Z* = 1, cluster extent = 10 voxels) (Radua et al., 2012).

To convert the SDM results into images, we used MRIcron software (http://www.mricro.com/mricron/), and overlaid the results on a high-resolution brain image template (created by the International Consortium for Brain Mapping) and the FMRIB58_ FA skeleton.

We then used DSI Studio to identify and visualize the WM tracts most probably involved, working as described in the DSI studio tutorial (http://dsi-studio.labsolver.org). Meta-analysis results were projected onto a high-resolution diffusion magnetic resonance imaging dataset generated from 80 subjects of the Human Connectome Project (Van Essen et al., 2012). A three-dimensional atlas of human white matter tracts (Catani and Thiebaut de Schotten, 2012) was used to identify the implicated tracts.

### Meta-Regression Analysis

Clinical variables explored by meta-regression analyses were mean BMI, age, Beck Depression Inventory (BDI) and illness duration, and percentages of females, and medicated patients. As in previous meta-analyses (Jiang et al., 2017) and in accordance with the recommendations of SDM's authors (Radua and Mataix-Cols, 2009), we adopted a conservative threshold of *p* = 0.0005 to minimize Type I error.

## Results

### Description of Included Studies

**Figure 1:**
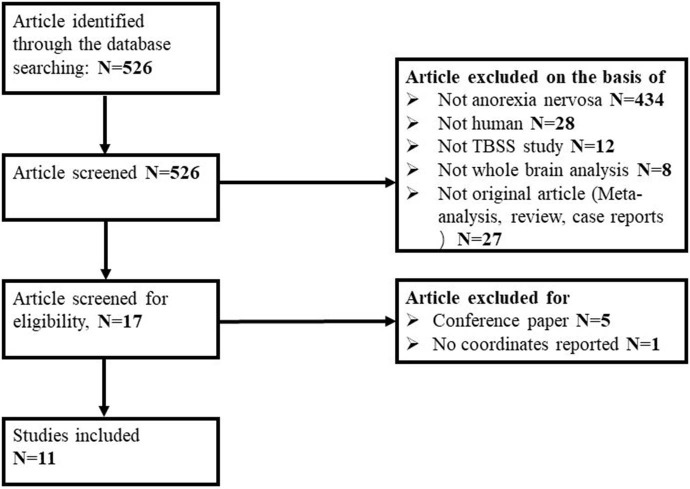
Flow diagram for the identification and exclusion of studies.

Of 526 potentially relevant studies, 11 met our criteria, as summarized in Figure 1. The 11 included studies recruited a total of 245 AN patients and 246 HC. Table 1 summarizes the clinical and demographic data from all included studies. The clinical characteristics (age, sex) of these studies showed no differences between AN and HC groups. Table 2 summarizes technical details of all included studies.

**Table 1:** Demographic and clinical characteristics of the participants in the 11 studies on anorexia nervosa included in the meta-analysis.

**Study**	**AN** **stage**	**AN** **subtype**	**Number (female)**		**Age (y)**		**Duration**	**BMI**		**BDI** **score**	**Onset age** **(y)**	**Medication %**	**Comorbidity**
			**AN**	**HC**	**AN**	**HC**	**(y)**	**AN**	**HC**				
Yau et al. (2013)	REC	R	12 (12)	10 (10)	28.7	26.7	5.6	21.2	22.0	NA	15.5	Drug-free	No
Nagahara et al. (2014)	A	R and B/P	17 (17)	18 (18)	23.8	26.2	4.93	13.6	19.9	30.2	NA	35	Yes
Via et al. (2014)	A	R	19 (19)	19 (19)	28.3	28.6	6.52	17.0	21.1	NA	21.8	26	Yes
Shott et al. (2016)	REC	R	24 (24)	24 (24)	30.3	27.4	5.90	20.8	21.6	NA	16.7	25	Yes
Cha et al. (2016)	A	R and B/P	22 (22)	18 (18)	19.5	20.5	NA	17.3	21.2	NA	NA	Drug-free	Yes
Olivo et al. (2017)	A	EDNOS	12 (12)	14 (12)	15.3	14.1	NA	18.7	20.6	NA	NA	Drug-free	Yes
Bang et al. (2018)	REC	R and B/P	21 (21)	21 (21)	27.6	26.1	2.83	20.5	21.8	6.6	17.3	14	No
Gaudio et al. (2017)	A	R	14 (14)	15 (15)	15.7	16.3	0.41	16.2	21.1	30.4	15.4	Drug-free	No
Phillipou et al. (2018)	A	R and B/P	23 (23)	26 (26)	22.0	22.6	5.35	16.7	22.8	NA	16.4	NA	Yes
von Schwanenflug et al. (2019)	A	R	56 (56)	56 (56)	15.9	16.2	1.21	14.7	20.6	21.2	NA	2	No
Olivo and Swenne (2019)	A	Atypical	25 (25)	25 (25)	14.8	14.5	0.7	18.6	20.0	NA	NA	Drug-free	No

*A, accurate; AN, anorexia nervosa; BDI, Beck Depression Inventory; BMI, body mass index; B/P: binge and purge subtype of anorexia nervosa; HC, healthy controls; NA, not available; R, restrictive subtype of anorexia nervosa; REC, recovered; EDNOS, eating disorder not otherwise specified*.

**Table 2:** Technical details of the 11 studies on anorexia nervosa included in the meta-analysis.

**Study**	**MRI scanner**	**No. of DTI** **directions**	**Coordinate** **system**	**Analysis** **software**	**Analysis** **method**	***p*-value**	**No. of coordinates**
Yau et al. (2013)	3.0T	55	MNI	FSL	TBSS	*p* < 0.05 (FWE)	0
Nagahara et al. (2014)	3.0T	32	MNI	FSL	TBSS	*p* < 0.08 (corrected)	1
Via et al. (2014)	1.5T	25	MNI	FSL	TBSS	*p* < 0.05 (FWE)	1
Shott et al. (2016)	NA	25	MNI	FSL	TBSS	*p* < 0.05 (FWE)	6
Cha et al. (2016)	1.5T	16	MNI	FSL	TBSS	*p* < 0.05 (FDR)	0
Olivo et al. (2017)	3.0T	48	MNI	FSL	TBSS	*p* < 0.05 (FDR)	2
Bang et al. (2018)	3.0T	32	MNI	FSL	TBSS	*p* < 0.05 (FWE)	0
Gaudio et al. (2017)	1.5T	12	MNI	FSL	TBSS	*p* < 0.05 (FWE)	4
Phillipou et al. (2018)	3.0T	60	MNI	FSL	TBSS	*p* < 0.05 (FWE)	1
von Schwanenflug et al. (2019)	3.0T	32	MNI	FSL	TBSS	*p* < 0.05 (FWE)	1
Olivo and Swenne (2019)	3.0T	48	MNI	FSL	TBSS	*p* < 0.05 (TFCE)	0

*DTI, diffusion tensor imaging; FDR, false discovery rate; FSL, functional MRI of the brain (FMRIB) software library; FWE, family-wise error; MNI, Montreal Neurological Institute Space; NA, not available; TBSS, tract-based spatial statistical; TFCE, threshold-free cluster enhancement*.

### Meta-Analysis

#### Pooled Voxel-Based Meta-Analysis

**Figure 2:**
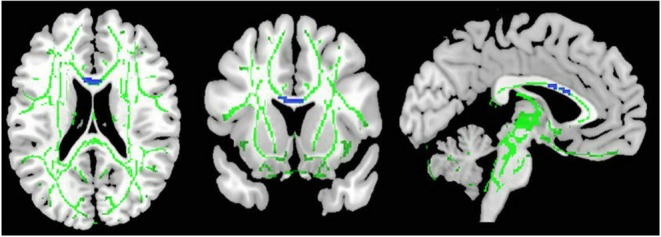
Results of pooled meta-analysis. Regions with blue color show lower FA in AN patients compared with healthy controls in the CC. FA, fractional anisotropy; AN, Anorexia nervosa; CC, Corpus callosum.

**Figure 3:**
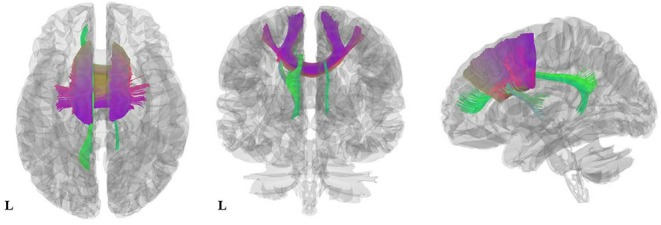
Results of pooled meta-analysis. Three-dimensional images show the most probable white matter tracts running through CC (purple) and the cingulum (green) bundle in AN patients. AN, Anorexia nervosa; CC, Corpus callosum.

As illustrated in Figure 2 and Table 3, the pooled meta-analysis revealed significantly *lower* FA in AN patients relative to HC in two regions: CC and cingulum. No regions showed *higher* FA. As shown in Figure 3, the WM tracts mainly involved were the cingulum bundle and the interhemispheric fibers running through the CC.

**Table 3:** Regions of lower fractional anisotropy in anorexia nervosa patients compared with health controls identified by the main meta-analyses.

**Region**	**Maximum**				**Cluster**	**Robustness**		
	**MNI coordinates** **x, y, z**	**SDM** **z-score**	***P*-value** **uncorrected**	**Number of** **voxels**	**Breakdown** **(No. of voxels)**	**Jackknife**	**Heterogeneity**	**Publication bias**
Corpus callosum	0, 6, 24	−2.070	~0	289	Corpus callosum (275)	10 of 11	0.00001	ns
					Cingulum (14)			

*MNI, Montreal Neurological Institute Space; SDM, Seed-based d Mapping; ns, non-significant; Jackknife: The jackknife sensitivity analysis column gives the number of studies whose omission does not affect the finding*.

#### Subgroup Voxel-Based Meta-Analysis

The adult AN subgroup included seven datasets that showed *lower* FA in the CC and cingulum bundle, sharing same clusters with the pooled meta-analysis. No regions showed *higher* FA in adult AN (Table 4).

**Table 4:** Regions of lower fractional anisotropy in anorexia nervosa patients compared with health controls identified by the subgroup meta-analyses (acute subgroup; adult subgroup).

**Region**	**Maximum**				**Cluster**	**Jackknife sensitivity analysis**
	**MNI coordinates** **x, y, z**	**SDM** **z-score**	***P*-value** **uncorrected**	**Number of** **voxels**	**Breakdown** **(No. of voxels)**	
**Acute subgroup**						
Corpus callosum	−6, 16, 20	−2.112	0.000024557	284	Corpus callosum (270)	8 of 8
					Cingulum (14)	
**Adult subgroup**						
Corpus callosum	−6, 14, 22	−1.071	0.000147164	138	Corpus callosum (127)	6 of 7
					Cingulum (11)	

*MNI, Montreal Neurological Institute Space; SDM, Seed-based d Mapping*.

The acute AN patient subgroup included 8 datasets that showed *lower* FA in the CC and cingulum bundle. No regions showed *higher* FA in AN (Table 4). These results are consistent with the pooled meta-analysis, indicating that the main effects related to the acute AN patients rather than recovered AN.

#### Reliability Analysis

The whole-brain jack-knife sensitivity analysis showed that *lower* FA in the CC and cingulum was highly reliable, being retained throughout 10 datasets combinations (Table 3). Analysis of heterogeneity revealed that the CC and cingulum with lower FA had significant statistical heterogeneity among studies (*p* < 0.005) (Table 3). Analysis of publication bias by the Egger test was non-significant for CC (*p* = 0.137) and cingulum (*p* = 0.484) (Table 3).

### Meta-Regression Analysis

Mean age and BMI showed no relationship with lower FA. Illness duration, BDI score and percentage of medicated patients could not be examined because of limited data. AN symptom severity could not be examined because it was reported using various inconsistent measures.

## Discussion

This is the first quantitative meta-analysis integrating TBSS studies in patients with AN. Partly consistent with our hypotheses, pooled analysis revealed that the most robust disruption of WM microstructure, reflected in lower FA, in AN patients were in the CC and cingulum. Subgroup analyses of adult studies and acute studies replicated these findings. However, we found no significant correlations between BMI and lower FA.

### Lower Fractional Anisotropy in the Corpus Callosum and Cingulum Bundle

The biggest cluster with lower FA in AN was the CC, as reported in several studies (Frieling et al., 2012; Frank et al., 2013; Shott et al., 2016; Gaudio et al., 2017; Olivo et al., 2017; Phillipou et al., 2018; Barona et al., 2019; von Schwanenflug et al., 2019). The CC is the largest interhemispheric commissure, communicating perceptual, cognitive, motor and affective information (Hofer and Frahm, 2006; Catani and Thiebaut de Schotten, 2008). Notably, the WM fibers crossing through the body of the CC connect the bilateral prefrontal cortices and supplementary motor areas (SMA), and microstructural alterations in the body of the CC, as reflected by the decreased FA, might lead to reduced quantity and speed of information transfer between these brain areas. The prefrontal cortices are involved in the affective element of body image, which can be conceptualized as feelings and the satisfaction or dissatisfaction with the body (Gaudio and Quattrocchi, 2012). Therefore, the lower FA in the body of CC in AN might reflect an impaired prefrontal interhemispheric connectivity, underlying or contributing to body image distortion in AN (Gaudio et al., 2014; Gadsby, 2017). The SMA is involved in the planning and control of motor actions, and plays an important role in task switching, especially in proactive behavioral switching (Nachev et al., 2008; Hikosaka and Isoda, 2010). Functional MRI has shown that the SMA is consistently activated when subjects switch between two tasks proactively in response to a cue (Rushworth et al., 2002). Therefore, we speculated that impaired WM integrity in the bilateral SMA might lead to cognitive-behavioral inflexibility (i.e. stereotyped or perseverative behaviors), which may contribute to behaviors for self-induced starvation. Furthermore, the observation that higher FA in the body of CC is positively correlated with reward-related activation in the nucleus accumbens suggests that CC might influence reward responsiveness of the ventral striatum by regulating the efficiency of information transfer within reward-related circuitries (Koch et al., 2014).

We also identified lower FA in the cingulum, in line with prior studies (Kazlouski et al., 2011; Frank et al., 2013). The cingulum incorporates fibers of different length: the longest running from the anterior temporal gyrus to the orbitofrontal cortex, while short *U*-shaped fibers link the medial frontal, parietal, occipital, and temporal lobes and different parts of the cingulate cortex (Catani and Thiebaut de Schotten, 2008). The cingulum is a component of the limbic system, involved in attention, memory and emotions (Catani, 2006; Rudrauf et al., 2008). Given that the cingulum bundle is a key part of the network integrating behaviors necessary for emotion identification and processing (Kazlouski et al., 2011), disruption of WM microstructures in this area could explain abnormalities in emotion recognition and regulation in AN, such as difficulties in concentrating and accomplishing tasks when experiencing negative emotions (Harrison et al., 2010).

Interestingly, these results of lower FA in the CC and cingulum were retained in the subgroup meta-analysis. The findings seem to show that the CC and cingulum are stable markers of the disorder and interruptions in WM tracts of these areas may be involved in the pathological mechanisms of AN. As the numbers of studies in the subgroup meta-analyses are relatively small (seven and eight respectively), we should treat these results with caution. Additionally, because limited data precluded meta-analysis of the recovered AN group, whether or not the alterations persist after recovery is a question still to be addressed.

### Null Results by Meta-Regression Analysis

Although there were no significant associations between clinical variables and WM abnormalities, the effect of self-starvation (*viz*. decreased BMI) is particularly interesting. Previous studies have variously reported significant correlations (Kazlouski et al., 2011; Nagahara et al., 2014; Olivo et al., 2017) and no correlations (Gaudio et al., 2017; Bang et al., 2018; Phillipou et al., 2018) between BMI and FA in different brain areas. Heterogeneity in patient characteristics may contribute to this negative result. Alternatively, it may indicate that WM microstructure impairments in AN are not directly related to effects of starvation, but instead to trait characteristics of the disorder (Phillipou et al., 2018). Nevertheless, these preliminary findings need to be validated by longitudinal studies.

### An Unexpected Lack of Abnormality

Compared with a previous meta-analysis of AN, which revealed disturbed WM in various regions (e.g. clusters with lower FA in the left superior longitudinal fasciculus and left precentral gyrus, and higher FA in the right cortico-spinal projections and lingual gyrus) (Barona et al., 2019), the present meta-analysis predominantly emphasized interhemispheric communication and the limbic association fibers. This inconsistency might be explained in two ways. Firstly, we only analyzed DTI studies using TBSS, not VBA, thus avoiding any bias arising from methodological differences in diffusion data processing. Secondly, we included a number of new studies, with resulting differences in sample characteristics (e.g., age, gender, subtype, and medication status).

### Limitations of This Study

The study has some limitations. Firstly, voxel-based meta-analyses are based on summarized data (i.e. coordinates and effect sizes from published studies). Although analyzing a cumulative set of primary data would in theory yield more accurate results, it is rarely feasible to obtain raw image files. Secondly, we could not take AN-subtypes into consideration; the restricting subtype and the binge-purging subtype may have different etiologies, but this was hard to explore because the information was not available. Thirdly, although we found the lower FA in the CC and cingulum retained significance in adult AN and acute AN subgroup analyses, it cannot be concluded that these abnormalities are a biomarker of the disorder, since the differences in their comparator groups (recovered AN and adolescent AN) are still unknown. More studies on these subgroups are needed. Fourthly, the mean FA skeleton is different in each study due to heterogeneity in the data, which may decrease the accuracy of the results of the meta-analysis. Finally, it is useful to combine FA with other diffusion parameters (MD, AD, and RD); unfortunately, most of the included studies did not report them.

## Conclusion

This meta-analysis detected significantly lower FA in AN in the WM of the interhemispheric connection and limbic association fibers, which are involved in body cognitive-behavioral inflexibility, image processing and emotional function. Although the neuropathology of AN is complex, our findings help provide evidence on how symptoms and behaviors are encoded in the brain, and thus may aid in developing effective treatments. Future studies with a longitudinal approach are needed to confirm our results and to reveal the trajectory of the pathophysiology.

## Data Availability Statement

All datasets generated for this study are included in the article/Supplementary Material.

## Author Contributions

QG and QY conceived the project. HS designed the protocol. SZ and WW wrote the main manuscript. SZ, WW, XS, XY, JS, and QT obtained the data. SZ, LL, and YZ analyzed the results. All authors critically reviewed the manuscript. GK, QG, and QY revised the manuscript.

### Conflict of Interest

The authors declare that the research was conducted in the absence of any commercial or financial relationships that could be construed as a potential conflict of interest.
