# Supplementary material for: White Matter Abnormalities in Anorexia Nervosa: Psychoradiologic Evidence From Meta-Analysis of Diffusion Tensor Imaging Studies Using Tract Based Spatial Statistics
Source: Front Neurosci. 2020 Mar 3;14:159. doi: 10.3389/fnins.2020.00159 (PMC7063983; doi:10.3389/fnins.2020.00159)
Supplement: Supplementary file 1 [file Table_1.docx]

| **Table S1. The quality assessment checklist and the scores of the included studies** |  |  |  |  |  |  |  |  |  | |  |  |
| --- | --- | --- | --- | --- | --- | --- | --- | --- | --- | --- | --- | --- |
| **Score (0/0.5/1)** | Yau | Nagahara | Via | Shott | Cha | Olivo | Bang | Gaudio | Phillipou | | von Schwanenflug | Olivo |
| **Category 1: Participants** |  |  |  |  |  |  |  |  | |  |  |  |
| 1 Patients were evaluated prospectively, specific diagnostic criteria were applied, and demographic data were reported | 1 | 1 | 1 | 1 | 1 | 1 | 1 | 1 | | 1 | 1 | 1 |
| 2 Healthy comparison participants were evaluated prospectively; psychiatric and medical illnesses were excluded | 1 | 1 | 1 | 1 | 1 | 1 | 1 | 1 | | 1 | 1 | 1 |
| 3 Important variables (e.g., age, gender, medication status, comorbidity, and subtype) were checked either via stratification or statistics | 1 | 1 | 1 | 1 | 0.5 | 0.5 | 1 | 1 | | 0.5 | 1 | 0.5 |
| 4 Sample size per group: ≥ 20 scores 1, ≥ 10 scores 0.5 | 0.5 | 0.5 | 0.5 | 1 | 0.5 | 0.5 | 1 | 0.5 | | 1 | 1 | 1 |
| **Category 2: Methods for image acquisition and analysis** |  |  |  |  |  |  |  |  | |  |  |  |
| 5 Magnet strength at least 1.5T | 1 | 1 | 1 | 1 | 1 | 1 | 1 | 1 | | 1 | 1 | 1 |
| 6 MRI slice-thickness≤3 mm | 1 | 1 | 1 | 1 | 1 | 1 | 1 | 1 | | 1 | 1 | 1 |
| 7 Whole brain analysis was automated with no a-priori regional selection | 1 | 1 | 1 | 1 | 1 | 1 | 1 | 1 | | 1 | 1 | 1 |
| 8 Coordinates reported in a standard space | 1 | 1 | 1 | 1 | 1 | 1 | 1 | 1 | | 1 | 1 | 1 |
| 9 The imaging technique used was clearly described so that it could be reproduced | 1 | 1 | 1 | 1 | 1 | 1 | 1 | 1 | | 1 | 1 | 1 |
| 10 Measurements were clearly described so that they could be reproduced | 1 | 1 | 1 | 1 | 1 | 1 | 1 | 1 | | 1 | 1 | 1 |
| **Category 3: Results and conclusions** |  |  |  |  |  |  |  |  | |  |  |  |
| 11 Statistical parameters for significant, and important non-significant, differences were provided | 1 | 1 | 1 | 1 | 1 | 1 | 1 | 1 | | 1 | 1 | 1 |
| 12 Conclusions were consistent with the results obtained and the limitations were discussed | 1 | 1 | 1 | 1 | 1 | 1 | 1 | 1 | | 1 | 1 | 1 |
| **Total score** | 11.5 | 11.5 | 11.5 | 12 | 11 | 11 | 12 | 11.5 | | 11.5 | 12 | 11.5 |
